# The dynamics of the piglet gut microbiome during the weaning transition in association with health and nutrition

**DOI:** 10.1186/s40104-018-0269-6

**Published:** 2018-07-30

**Authors:** Robin B. Guevarra, Sang Hyun Hong, Jin Ho Cho, Bo-Ra Kim, Jiwon Shin, Jun Hyung Lee, Bit Na Kang, Young Hwa Kim, Suphot Wattanaphansak, Richard E. Isaacson, Minho Song, Hyeun Bum Kim

**Affiliations:** 1 Department of Animal Resources Science, Dankook University, Cheonan, South Korea; 2 Division of Food and Animal Sciences, Chungbuk National University, Cheongju, South Korea; 3 Abbvie Bioresearch Center, Abbvie, Worcester, MA USA; 4 National Institute of Animal Science, Rural Development Administration, Wanju, South Korea; 5 Department of Veterinary Medicine, Faculty of Veterinary Science, Chulalongkorn University, Pathum Wan, Bangkok, 10330 Thailand; 6 Department of Veterinary and Biomedical Sciences, University of Minnesota, St. Paul, MN 55108 USA; 7 Division of Animal and Dairy Science, Chungnam National University, Daejeon, South Korea

**Keywords:** Metagenomics, Microbiome, Piglets, 16S rRNA, Weaning

## Abstract

**Background:**

Understanding the composition of the microbial community and its functional capacity during weaning is important for pig production as bacteria play important roles in the pig’s health and growth performance. However, limited information is available regarding the composition and function of the gut microbiome of piglets in early-life. Therefore, we performed 16S rRNA gene and whole metagenome shotgun sequencing of DNA from fecal samples from healthy piglets during weaning to measure microbiome shifts, and to identify the potential contribution of the early-life microbiota in shaping piglet health with a focus on microbial stress responses, carbohydrate and amino acid metabolism.

**Results:**

The analysis of 16S rRNA genes and whole metagenome shotgun sequencing revealed significant compositional and functional differences between the fecal microbiome in nursing and weaned piglets. The fecal microbiome of the nursing piglets showed higher relative abundance of bacteria in the genus *Bacteroides* with abundant gene families related to the utilization of lactose and galactose. *Prevotella* and *Lactobacillus* were enriched in weaned piglets with an enrichment for the gene families associated with carbohydrate and amino acid metabolism. In addition, an analysis of the functional capacity of the fecal microbiome showed higher abundances of genes associated with heat shock and oxidative stress in the metagenome of weaned piglets compared to nursing piglets.

**Conclusions:**

Overall, our data show that microbial shifts and changes in functional capacities of the piglet fecal microbiome resulted in potential reductions in the effects of stress, including dietary changes that occur during weaning. These results provide us with new insights into the piglet gut microbiome that contributes to the growth of the animal.

**Electronic supplementary material:**

The online version of this article (10.1186/s40104-018-0269-6) contains supplementary material, which is available to authorized users.

## Background

The mammalian gastrointestinal tract (GIT) harbors 500–1000 bacterial species that play important roles in the health and disease of the host [1]. It is known that early bacterial gut colonizers are important in the initial establishment of the complex gut microbial community. The gut microbiome is thought to play many important roles in the health and growth of animals including the reduction in the incidence of infectious, inflammatory, and other immune diseases [2, 3] and contributing to the overall metabolism and, therefore, the growth of the animal.

Weaning is a stressful event in a pig’s life and can disrupt the piglet gut microbiome, which can lead to poor health and growth performance [4]. Piglets experience a wide variety of stresses such as physiological, environmental and social challenges during the weaning transition [4]. This is important to the swine industry since the changes in the composition of the gut microbiota after weaning can lead to an increased susceptibility of piglets to post-weaning diarrhea. Consequently, this can lead to an economic burden for pig farmers [5]. During the weaning transition, the diet of piglets abruptly shifts from a high-fat, low-carbohydrate milk to a high-carbohydrate and low-fat feed. This change may lead to reduced proliferation of intestinal epithelial cells [6]. Previously, it has been reported that the diet shapes the gut microbiome of piglets during nursing and weaning periods [7]. More recently, the relationship between body weight and intestinal microbiota in weaned piglets has also been investigated [8]. It has been shown that the introduction of solid feed and the weaning process are important driving forces in the succession of gut bacteria in piglets [9]. Even though, these studies have emphasized the great importance of early-life microbiota to growth, immune system development and the health of piglets, there is limited information available regarding the structure and function of the gut microbiome of piglets in early-life in association with health and growth performance.

Antimicrobial growth promoters (AGPs) have been used in swine production for several decades. However, their use has been banned in many countries worldwide because of potential side effects, such as emergence of resistance to antimicrobials. Thus, efforts to develop alternatives to AGPs with the goal of preserving the efficacy of AGPs are being implemented. In this sense, developing alternate ways to promote growth makes it more important to understand microbial and functional succession of the piglet gut microbiome because one of the specific mechanisms of AGPs is to alter gut microbial population composition [10, 11].

Therefore, the work described in this study was designed to better document the changes that occur in the composition of the fecal microbiome in piglets before and after weaning and to use metagenomics to identify metabolic functions that may be changed during these time points. It is a goal of this study to bring new insights into the potential contribution of early-life microbiota in shaping the host metabolism and health with focus on stress response, carbohydrate and amino acid metabolism.

## Methods

### Fecal sampling

Fecal samples were collected from the rectum of 10 piglets just prior to weaning (21 d of age) and again 1 wk after weaning (28 d of age). The fecal samples were placed in sterile test tubes and stored at − 80 °C. After weaning, the piglets were fed a typical nursery diet based on corn and soybean meal. The diet was formulated to meet the National Research Council [12] estimates of nutrient requirements of weaned piglets (Table 1). Piglets were allowed free access to feed and water. No antibiotics or supplementary additives were administered to the piglets throughout the experiment.

**Table 1:** Composition of basal diet for weaned pigs (as-fed basis)

Item	Content
Ingredient, %	
Corn	56.09
Soybean meal, 44%	26.00
Soy protein concentrate	12.00
Soybean oil	3.00
Limestone	1.30
Monocalcium phosphate	1.20
Vit-Min premix^1^	0.04
*L*-Lysine HCl	0.24
*DL*-Methionine	0.09
*L*-Threonine	0.04
Total	100
Calculated energy and nutrient contents	
ME, Mcal/kg	3.48
CP, %	24.17
Calicum, %	0.84
Phosphorus, %	0.66
Lysine, %	1.54
Methionine, %	0.45
Cysteine, %	0.39
Threonine, %	0.96
Tryptophan, %	0.28
Arginine, %	1.60
Histidine, %	0.67
Isoleucine, %	1.03
Leucine, %	2.05
Phenylalanine, %	1.21
Valine, %	1.09

^1^Provided per kilogram of diet: vitamin A, 12,000 IU; vitamin D_3_, 2500 IU; vitamin E, 30 IU; vitamin K_3_, 3 mg; *D*-pantothenic acid, 15 mg; nicotinic acid, 40 mg; choline, 400 mg; and vitamin B_12_, 12 μg; Fe, 90 mg from iron sulfate; Cu, 8.8 mg from copper sulfate; Zn, 100 mg from zinc oxide; Mn, 54 mg from manganese oxide; I, 0.35 mg from potassium iodide; Se, 0.30 mg from sodium selenite

### DNA extraction

Total DNA from the feces was extracted from 200 mg of feces per sample using QIAamp Fast DNA Stool Mini Kit (QIAGEN, Hilden, Germany) according to the manufacturer’s instructions. Cell lysis was performed by bead-beating the samples twice for 2 min at 300 r/min, with an incubation period of 5 min in a water bath at 70 °C between beatings. The concentrations of DNA were measured using a Colibri Microvolume Spectrometer (Titertek Berthold, Pforzheim, Germany) and samples with OD260/280 ratios of 1.80–2.15 were processed further.

### 16S rRNA gene and whole metagenome sequencing

For the 16S rRNA gene sequencing, the primers 799F-mod6 (5´-CMGGATTAGATACCCKGGT-3′) and 1114R (5´-GGGTTGCGCTCGTTGC-3′) were used to amplify the V5 through V6 hypervariable regions of the 16S rRNA gene [13]. The amplification mix contained 5× PrimeSTAR Buffer (Mg^2+^) (Takara Bio, Inc., Shiga, Japan), 2.5 mmol/L concentrations of each of deoxynucleotide triphosphates (dNTPs), 2.5 IU/μL of PrimeSTAR HS DNA Polymerase, a 10 pmol of each primer, and 25 ng of DNA in a reaction volume of 50 μL. The thermal cycling parameters were as follows: initial denaturation at 98 °C for 3 min, followed by 35 cycles of 98 °C for 10 s, 55 °C for 15 s, and 72 °C for 30 s, and a final 3-min extension at 72 °C. PCR products were purified using PCR purification kit, Wizard® SV Gel and PCR Clean-Up System (Promega, Wisconsin, USA). The barcoded16S rRNA gene amplicons were sequenced using the Illumina MiSeq platform at Macrogen Inc. (Seoul, Republic of Korea). For the whole metagenome shotgun sequencing, DNA representing the fecal microbial communities extracted from the feces was sequenced using paired-end shotgun sequencing using the Illumina Hi-Seq 2000 platform at Macrogen Inc. (Seoul, Republic of Korea).

### 16S rRNA gene sequence analysis

The 16S rRNA gene sequences were processed using the Mothur software to remove low-quality sequences [14]. Briefly, sequences that did not match the PCR primers were eliminated from demultiplexed sequence reads. We also trimmed sequences containing ambiguous base calls and sequences with a length less than 100 bp to minimize the effects of random sequencing errors. Chimeric sequences were further deleted using the UCHIME algorithm implemented in Mothur. QIIME (Quantitative Insights into Microbial Ecology) software package (version 1.9.1) was used for de novo operational taxonomic unit (OTU) clustering with an OTU definition at an identity cutoff of 97% [15]. Taxonomic assignment was performed using the naïve Bayesian RDP classifier and the Greengenes reference database. Microbial alpha diversity including Chao1, observed OTUs, phylogenetic diversity (PD) whole tree, Shannon index and Simpson index were calculated using QIIME. A two-sided Welch’s *t-*test in Statistical Analysis of Metagenomic Profiles (STAMP) software v2.1.3 [16] was used to identify significant differences in relative abundance of microbial taxa of the two groups. A *P* value < 0.05 was considered to be significant. Beta-diversity was measured using both weighted and unweighted UniFrac distance metrics using QIIME. The unweighted UniFrac takes into account the community membership (presence or absence of OTUs), whereas the weighted UniFrac considers the relative abundance of OTUs in the community [17]. Principal coordinate analysis (PCoA) plots were generated based on the weighted and unweighted UniFrac distance metrics. Analysis of similarities (ANOSIM) was used to determine whether the microbial compositions between the two groups were significantly different using QIIME and was based on the weighted and unweighted UniFrac distance metrics.

### Whole metagenome sequence analysis

Whole metagenome shotgun sequencing was performed on a subset of eight samples selected randomly (four samples from the same piglets at 21 and 28 d of age) to investigate the fecal microbial functions present in the fecal microbes of the samples. To analyze whole metagenomic sequence data from nursing and weaned piglets, the raw sequence data in FASTQ format were imported to the CLC Genomics Workbench (version 10) with CLC Microbial Genomics Module (version 1.2) (Qiagen Bioinformatics, Aarhus, Denmark). The quality of the sequences was assessed and high quality sequences were assembled using CLC’s De Novo assembly algorithm. The contigs were submitted to the Metagenomics Rapid Annotation using Subsystem Technology (MG-RAST) pipeline for microbial functional analysis. All the sequence reads were normalized in MG-RAST. The MG-RAST pipeline uses DESeq to analyse sequence count data, and to remove aspects of inter-sample variability caused by differences in sequencing depth of samples [18]. Removal of artificial duplicate reads and pig genomic DNA reads were performed using the MG-RAST pipeline [19]. The MG-RAST pipeline uses bowtie to remove sequence reads that match to the genome of the host [20]. To filter out host-derived metagenomic reads, we used the reference swine genome (*Sus scrofa*, NCBI v10.2) readily available in MG-RAST [19]. The functional annotation of the sequence reads was performed using the SEED Subsystems database, a collection of functionally related protein families [21]. Similarity search between sequence reads and the SEED databases was conducted by using an *E*-value of less than 1 × 10^− 5^, minimum identity of 60%, and a minimum alignment length of 15 amino acids for protein. Multiple *t*-tests were used to identify significant differences in functional profiles between nursing and weaned pigs using STAMP and GraphPad Prism version 7.00 (La Jolla, CA, USA).

## Results

### Microbial diversity of nursing and weaned piglets based on 16S rRNA gene data

Sequencing of the 16S rRNA genes in the fecal samples produced a total of 1,947,836 reads after quality-filtering, with a mean sequence number of 97,392 ± 49,139 reads per sample (Additional file 1: Table S1). The diversity of the microbial communities in the fecal samples decreased after weaning as measured using Shannon, Simpson, and Chao1 diversity indices (Table 2). However, the differences observed between the two groups were not statistically significant with the exception of the phylogenetic diversity (PD) whole tree index. The mean number of observed OTUs identified in the nursing group was 631.60 ± 168.36 and 543.80 ± 141.54 for the weaned piglet samples. The Shannon-Weaver index values showed highly diverse microbial communities in nursing (5.13 ± 0.85) and weaned piglets (4.58 ± 1.11). The PD whole tree index, a measure of biodiversity that assimilates phylogenetic difference between taxa, was significantly higher in nursing piglets compared to the weaned piglets (*P* < 0.05).

**Table 2:** Alpha diversity of the piglet gut microbiota using 16S rRNA gene sequences

Diversity index	Nursing	Weaned	*P*-value
PD whole tree	17.58 ± 2.94	13.03 ± 2.45	0.003
Shannon	5.13 ± 0.80	4.58 ± 1.06	0.223
Simpson	0.89 ± 0.07	0.82 ± 0.13	0.189
Chao1	1192.99 ± 324.63	1090.64 ± 287.64	0.463
Observed OTUs	631.56 ± 159.71	543.72 ± 134.26	0.237

Values are presented as mean ± SD (*n* = 10 per group)

*OTU* operational taxonomic unit

**Fig. 1:**
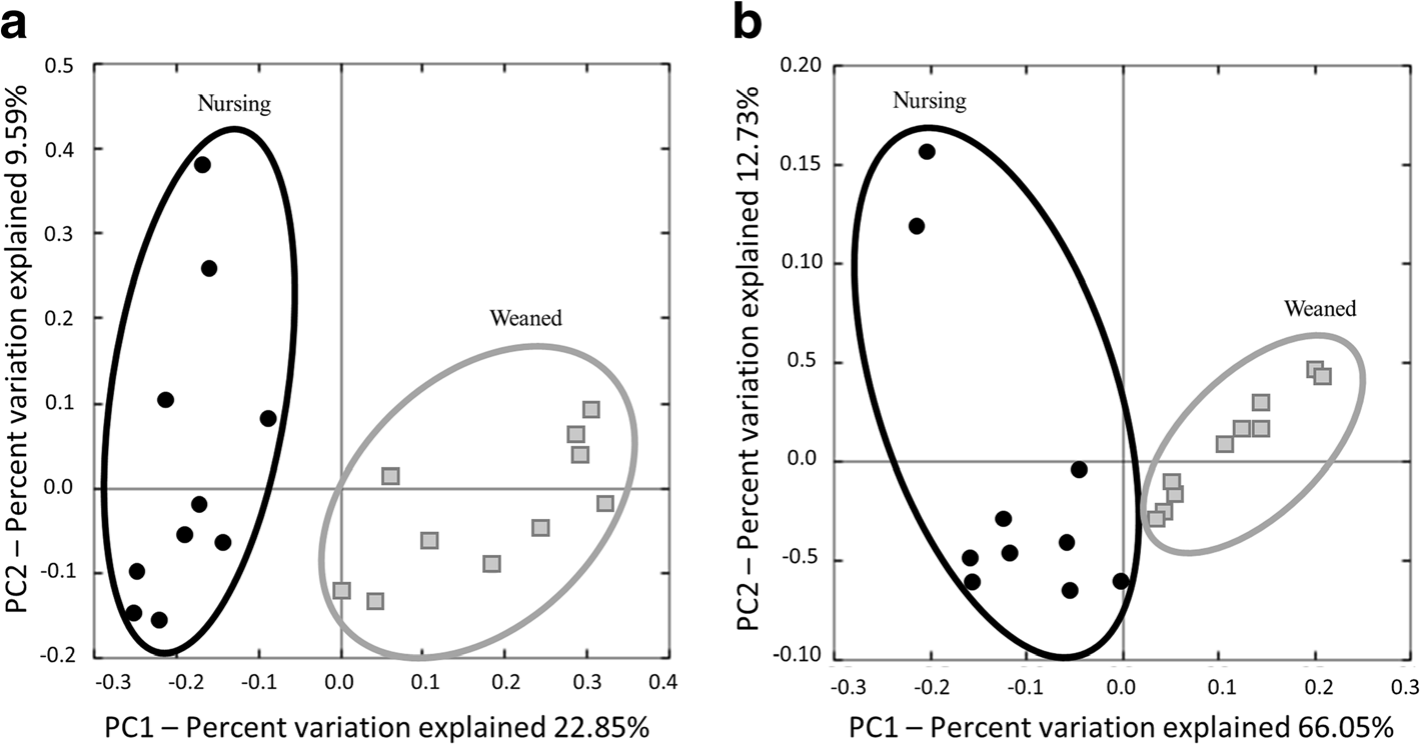
Principal coordinates analysis (PCoA) plots based on (**a**) unweighted and (**b**) weighted UniFrac distance metrics

Analysis of similarities (ANOSIM) of unweighted UniFrac distances indicated that nursing and weaned pigs were significantly different (*P* = 0.001) with relatively high *R*-value of 0.7373 suggesting that the microbiota of the two groups were significantly different. The unweighted UniFrac PCoA plot visually confirmed the distinct separation of microbial communities between the nursing and weaned piglets (Fig. 1a). The ANOSIM of weighted UniFrac distances were similar to the unweighted UniFrac distances, which showed a significant difference between the microbial communities of nursing and weaned pigs (*P* = 0.001) with an *R*-value of 0.7158. The PCoA plot of the weighted UniFrac distances also showed distinct clustering between nursing and weaned piglets (Fig. 1b).

### Taxonomic classification of the bacteria using 16S rRNA genes

**Fig. 2:**
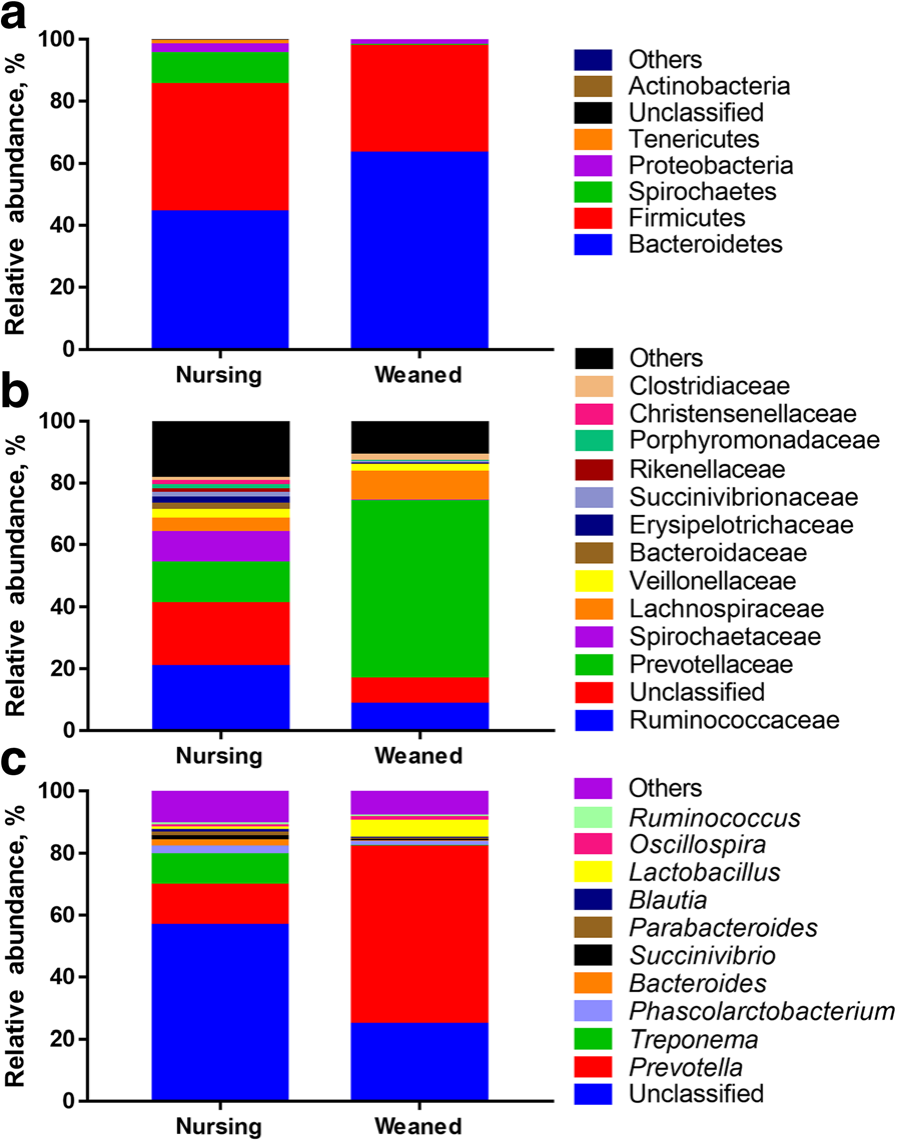
Taxonomic classification of the 16S rRNA gene sequences at the (**a**) phylum, (**b**) family, and (**c**) genus levels for the Nursing and Weaned piglets

Comparisons of the relative abundances of the gut microbiota compositions between nursing and weaned piglets at the phylum, family and genus levels are shown in Fig. 2. At the phylum level, the bacterial sequences from the nursing piglet samples were composed predominantly of the phyla Bacteroidetes (44.14%), Firmicutes (41.01%) Spirochaetes (9.87%), Proteobacteria (2.94%), Tenericutes (1.07%) and 14 other phyla that collectively comprised 0.61% of the total sequences analyzed (Fig. 2a). By comparison, weaned piglets consisted largely of phyla Bacteroidetes (63.14%), Firmicutes (34.27%), Proteobacteria (1.79%), Spirochaetes (0.29%), Tenericutes (0.26%) and other 14 phyla which collectively comprised of 0.05% of the total sequences analyzed in the weaned piglet samples (Fig. 2a). After weaning, the populations of the phylum Bacteroidetes significantly increased from an average of 44.14% in nursing pigs to 63.14% in weaned animals (*P* < 0.05). This coincided with a significant decrease in the populations of phyla Spirochaetes*,* Tenericutes, Actinobacteria, and Lentisphaerae (*P* < 0.05) (Fig. 2a).

At the family level, the three most abundant bacterial families in nursing pig microbiota primarily consisted of Ruminococcaceae (20.43%), Prevotellaceae (12.93%) and Spirochaetaceae (9.84%). After weaning, populations of Prevotellaceae and Lactobacillaceae significantly increased by 44.31% and 4.78%, respectively (Fig. 2b).

**Fig. 3:**
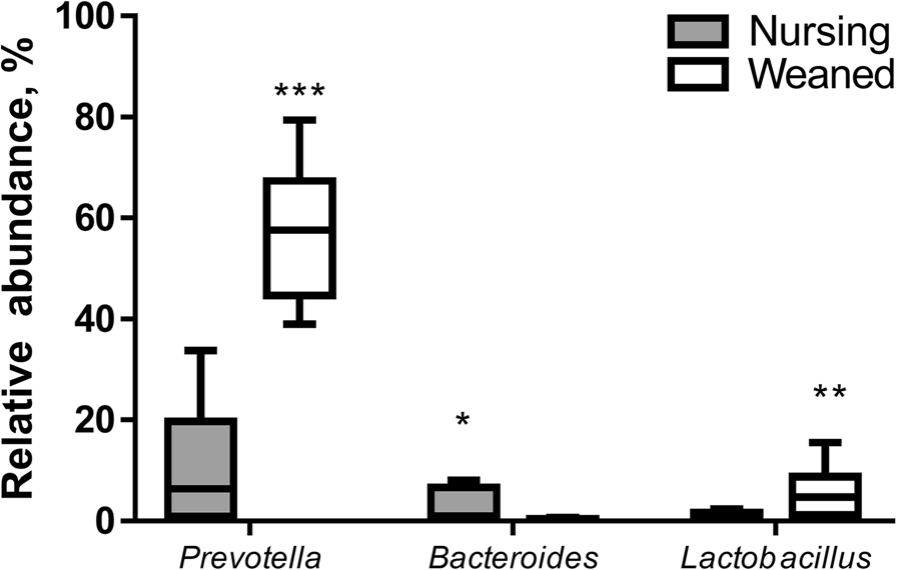
The Box plot identifying the significantly different taxa between Nursing and Weaned piglets at the genus level. The interquartile range is indicated by the outer bounds of the boxes, and the median is indicated by the black midline. The whiskers represent the minimum and maximum values. The [*P* < 0.001], [*P* < 0.01] and [*P* < 0.05] were indicated as [***], [**] and [*], respectively

At the genus level, *Prevotella* and *Lactobacillus* were the top 2 most significantly enriched genera in the weaned piglets while *Bacteroides* was the most abundant genera in nursing piglet fecal samples (*P* < 0.05) (Figs. 2c & 3). While *Prevotella* represented the most abundant genus in both groups, its relative abundance significantly increased (*P* < 0.001) from an average of 12.93% in nursing piglets to 57.24% in weaned piglets (Fig. 3). Similar to a previous report on the piglet gut microbiome, *Prevotella* was present in nursing piglets with a relatively low abundance and increased in weaned piglets when a plant-based diet was introduced [7]. The other genera that differ between nursing and weaned piglets are shown in Additional file 2: Figure S1.

The similar profiles of bacterial communities were observed when comparing the taxa between results obtained using the 16S rRNA gene data and whole metagenome shotgun sequencing (Additional file 2: Fig. S2).

### Microbial functional characteristics of the piglet gut metagenome associated with “stress response” and “virulence, disease and defense”

Overall, the whole metagenome shotgun sequencing using HiSeq Illumina platform produced a total of 50,440,732 sequences. After quality trimming, a total of 1,120,421 contigs were assembled from eight fecal samples (Additional file 1: Table S2). In the level 1 SEED subsystems, we identified 28 SEED Subsystems in both nursing and weaned piglet metagenome (Additional file 2: Figure S3), and we focused on the differences of functional gene groups associated with “carbohydrates”, “amino acids and derivatives”, “stress response”, and “virulence, disease, and defense” (Additional file 2: Figure S4).

**Fig. 4:**
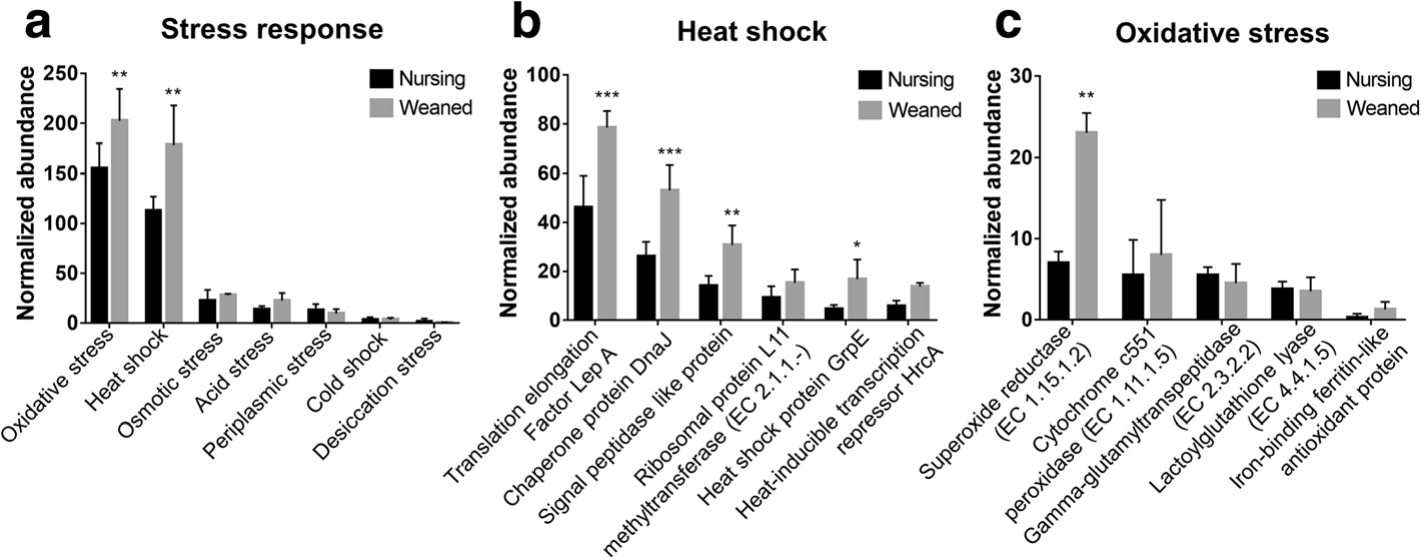
Comparison of the functional capacities of the gut microbiomes between Nursing and Weaned piglets associated with “stress response”. Normalized abundance of the level 2 SEED subsystem classified reads associated with stress response (**a**). Normalized abundance of proteins at the level 4 SEED subsystem associated with heat shock (**b**) and oxidative stress (**c**). The error bars show the calculated standard deviation of four replicates, and the [*P* < 0.001], [*P* < 0.01] and [*P* < 0.05] were indicated as [***], [**] and [*], respectively

Stresses and disturbances of the composition of the fecal microbiome during the weaning transition have been demonstrated to cause diarrhea and growth reduction [5]. Therefore, we investigated the impact of weaning on the functional profiles of the bacterial communities to evaluate counter responses of piglet gut microbiome against the stresses caused by weaning. At the level 2 SEED subsystems, within the “stress response”, gene families related to “oxidative stress” and “heat shock” were significantly enriched (*P* < 0.05) in the weaned piglets (Fig. 4a).

At the level 4 SEED subsystems within the “heat shock and oxidative stress”, numerous proteins and enzymes that were involved in bacterial heat shock and oxidative stress were significantly enriched (*P* < 0.05) in weaned piglets including translation elongation factor LepA, Chaperone protein DnaJ, signal peptidase-like protein, heat shock protein GrpE (Fig. 4b), superoxide reductase (EC1.15.1.2) and cytochrome c551 peroxidase (EC1.11.1.5) (Fig. 4c).

Diversity analysis of SEED subsystems retrieved from piglet gut microbial metagenome associated with “virulence, disease and defense” covered 2.45% of the total sequences assigned to SEED subsystems. Interestingly, at level 2 SEED subsystems, the most abundant gene family within the virulence, disease and defense was “resistance to antibiotics” while other functional gene groups such as “adhesion”, “detection” and “invasion and intracellular resistance” were less abundant (Additional file 2: Figure S5a). At the level 3 SEED subsystems, we observed that gene families associated with resistance to antibiotics have the tendency to be higher in weaned piglets including multidrug resistance efflux pumps, resistance to fluoroquinolones and beta-lactamase (Additional file 2: Figure S5b).

### Microbial functional characteristics of the piglet gut metagenome associated with carbohydrate and amino acid metabolism

**Fig. 5:**
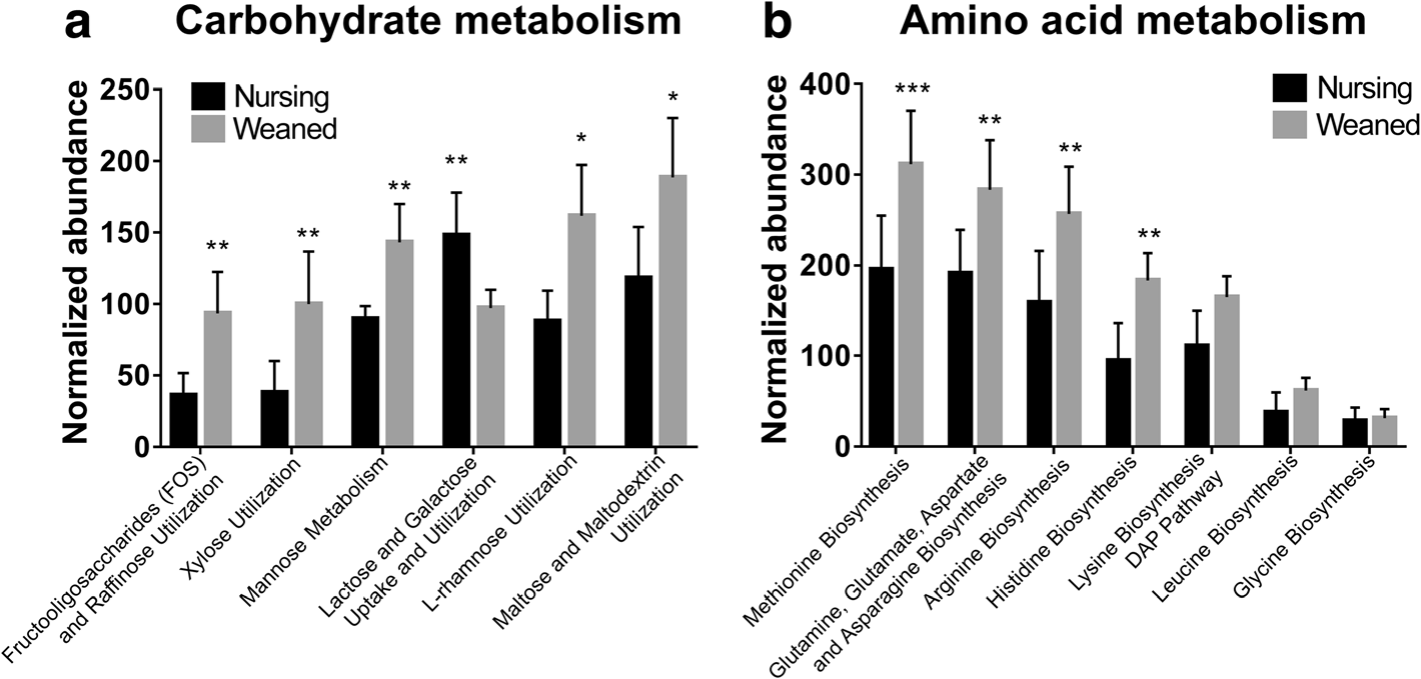
Comparison of the functional capacities of the gut microbiomes between Nursing and Weaned piglets associated with nutrition. Normalized abundance of the level 3 SEED subsystem classified reads associated with carbohydrate metabolism (**a**) and amino acid metabolism (**b**). The error bars show the calculated standard deviation of four replicates, and the [*P* < 0.001], [*P* < 0.01] and [*P* < 0.05] were indicated as [***], [**] and [*], respectively

We performed a hierarchical clustering-based analysis of the SEED subsystem and found that level 1 SEED subsystems associated with “carbohydrates” and “amino acids and derivatives” were significantly enriched in the weaned pigs (*P* < 0.05) (Additional file 2: Figures. S3 and S4). At the level 3 SEED subsystems, gene families mapping to carbohydrate and amino acid metabolism were significantly higher in the weaned piglets (Fig. 5).

The carbohydrate composition of the porcine diet abruptly changes when the pigs are separated from the sow and complex plant-based feeds are introduced. The composition and the functional capacity of the microbiota shift when such complex plant-derived glycans enter the gut. Similar to previous reports on the swine fecal metagenome [22, 23], we observed that the abundance of genes mapping to carbohydrates metabolism associated with components of plant-derived polysaccharides significantly more prevalent in weaned pigs including “xylose utilization”, “mannose metabolism” and “*L*-rhamnose utilization” (Fig. 5a). These sugars are products of the hydrolysis of non-starch polysaccharides (NSP) that are mainly found in many feed ingredients including soybean meal, wheat bran and oats. The microbiome of the nursing piglet had a significant enrichment of gene families associated with “lactose and galactose uptake and utilization” with lactose being the principal sugar in porcine milk (Fig. 5a). The relative abundance of genes associated with amino acid metabolism was also higher in weaned piglets than nursing piglets. Four of these pathways were significantly enriched in weaned piglets including “histidine biosynthesis”, “arginine biosynthesis”, “glutamine, glutamate, aspartate and asparagine biosynthesis” and “methionine biosynthesis” (*P* < 0.05) (Fig. 5b).

## Discussion

We performed this study to better understand microbial succession and changes in functional capacity of the piglet fecal microbiome during the weaning transition. One of the most striking observations in this study was the significant increase in the genus *Prevotella* after weaning. It has been reported that *Prevotella* is linked to the fermentation of plant-derived non-starch polysaccharides to short-chain fatty acids [24]. In humans, *Prevotella* spp. have also been reported to produce enzymes, such as β-glucanase, mannase, and xylanase that can degrade polysaccharides in plant cell wall [25]. The relative abundance of *Lactobacillus* also increased in weaned animals. *Lactobacillus* has been recently identified as bacteria with the ability to consume plant-derived monosaccharides and disaccharides [26]. The high abundance of *Lactobacillus* in the microbial community of weaned piglets is consistent with other studies on carbohydrate utilization in other mammalian species [27]. Generally, *Lactobacillus* is recognized as a carbohydrate-utilizing bacterium with numerous genes encoding a wide range of functional capacities associated with carbohydrate transport and utilization [28]. Our results suggest that *Lactobacillus* may also play a pivotal role in the utilization of complex carbohydrates. Overall, the present study reported higher abundances of *Prevotella* and *Lactobacillus* in the weaned piglets that may allow them to adapt to the dietary conditions after weaning.

It was noteworthy that the nursing piglet microbiota had significantly higher relative abundance of genus *Bacteroides,* which are well-known bacteria that utilize milk oligosaccharides as carbon sources [7]. Our analysis of the whole metagenome of nursing piglet microbiota showed significant enrichments of genes associated with lactose and galactose uptake and utilization. In comparison to human breast milk, porcine milk is primarily composed of lactose (Lac), glucose (Glc), galactose (Gal), N-acetyl-glucosamine (GlcNAc), fucose (Fuc), and sialic acids (NeuAc/ NeuGc) [29]. Milk oligosaccharides are composed of repeating units of lactose or N-acetyl-lactosamine that are usually bonded with sialic acid and fucose monosaccharides [30]. These complex milk oligosaccharides are not digested by the host during the passage through the GIT suggesting that they may play a role as natural prebiotics [31]. Our results suggest that both microbial composition and the metabolic functions of the nursing piglet microbiome is oriented to the utilization of milk oligosaccharides.

Weaning is a stressful event in a pig’s life and can contribute to intestinal and immune system dysfunctions [4]. Thus, understanding the microbial community structure and functional capacity of the microbiome during the weaning transition is substantial to pig production as it plays important roles in pig health and diseases. In this study, we found that there were significant enrichments of genes associated with bacterial heat shock responses in weaned pigs. The heat shock responses in bacteria are a result of a stress and are important for successfully adapting to changes in the physiological state, as well as to changes in the environment of the bacterial habitat [32]. Heat shock responses involve the induction of heat shock proteins (HSP) that are comprised of a set of well-conserved proteins with molecular mass ranging from 27 to > 100 kDa, and are produced by bacteria [33, 34]. HSP play a major role in the protection of cells by functioning as intra-cellular chaperones for other proteins under different kinds of stressors [34]. Even though the exact mechanisms of the HSP are yet to be determined, our data suggest that bacterial heat shock response in weaned piglets may play a role in mitigating the negative effects associated with weaning.

The present study also showed a significantly higher abundance of functional gene groups associated with oxidative stress response in the bacterial metagenome of the weaned piglet. Oxidative stress is defined as a disturbance in the balance between the production of reactive oxygen species (ROS) and antioxidant defenses [35]. The increase in production of ROS can cause damage to biological molecules including DNA, protein and lipids and can even lead to cell death [36]. A previous study indicated that weaning causes oxidative stress and the exposure of bacterial cells to oxidative stress can have damaging effects on protein activities and can contribute to death [37, 38]. As such, oxidative stress caused by weaning eventually induced enterocyte apoptosis and cell cycle arrest in the small intestine of post-weaning piglets [37]. A recent report on the impact of the gut microbiota on the development of metabolic diseases revealed that *Lactobacillus* spp. have developed defense mechanisms against oxidative stress [39]. The higher proportion of sequences that mapped to oxidative stress genes in weaned piglets appear to be reflected in the increase of the relative abundance of *Lactobacillus* observed in this study. However, potential roles of *Lactobacillus* spp. to regulate oxidative stress in weaned piglets should be validated through evidence-based experiments.

Amino acids play crucial physiological roles in young piglets to support their maximum production performance [40]. In this study, SEED subsystems related to amino acid metabolism were significantly elevated in the weaned piglets. These findings can be attributed to the increased use of amino acids in feed for protein accretion in livestock production. Although glucose is widely accepted as the primary nutrient for the maintenance and promotion of cell function, it has been reported that glutamate, glutamine and aspartate are the major contributors to the oxidative fuel for the intestine and cells of the immune system [41, 42]. Moreover, the present study showed that arginine biosynthesis was significantly enriched in the microbiome of weaned piglets. In addition, a recent study showed that arginine supplementation in weaned piglets has beneficial effects against oxidative stress in the jejunum through the suppression of inflammatory cytokine expression [43]. While the small intestine is the major site for amino acid absorption, the absorption of amino acids in the large intestine is limited [44]. However, amino acids in the colon, including lysine, arginine, glycine, leucine, valine and isoleucine can be used by the colonic bacteria to generate a complex mixture of metabolic products, such as short-chain fatty acids (SCFA), which are available energy source to the pigs [45]. Our results have provided us with new insights into the functional aspect of the microbiome to help us better understand the interplay between amino acid metabolism in bacteria and pig health in response to an abrupt dietary change that occurs during the weaning transition. Nevertheless, further studies are required to elucidate the exact roles of the functional gene groups of the weaned piglet gut microbiome associated with the amino acid metabolism for the swine performance.

## Conclusions

We observed distinct microbial communities and functional capacities of the piglet gut microbiome between nursing and weaned piglets. The weaning process significantly altered the composition and functional capacities of the gut microbiome. As such, our data suggest that the early-life stressors caused by dietary change could be an important driver to lead to these microbiome shifts. Even though further studies are required to elucidate the effect of microbiome shifts on piglet health, our data suggest that the microbiome shift and changes in functional capacities of the pig gut microbiome were oriented to deal with heat shock and oxidative stress during the weaning transition. Overall, our results suggest that piglets overcome stresses caused by dietary change during the weaning transition through a gut microbiome shift, and these results emphasize the importance of the early-life microbiota.

We believe that our results provide us with substantial insights into the piglet gut microbiome that contributes to the growth of the animal, and help us to better understand the important roles of the essential gut microbiome for later studies aiming to develop pig gut modulators, such as feed additives.

## Additional files

Additional file 1: **Table S1.** Number of 16S rRNA gene sequence reads of nursing and weaned piglet fecal microbiota before and after quality control. **Table S2.** Summary of whole metagenome sequence data before and after quality control and annotation. (DOCX 18 kb)

Additional file 2: **Figure S1.** Extended error bar plot identifying the significantly different taxa between nursing and weaned piglets at the phylum (a), family (b) and genus (c) levels. Corrected *P* values are shown at right. The differences in the microbial community structure were measured using a two-sided Welch’s t-test, and *P* < 0.05 was considered significant. **Figure S2.** Comparison of the taxonomic profiles obtained using 16S rRNA gene and whole metagenome sequencing between nursing and weaned piglets. Stacked bar plots show the relative abundance of bacteria at the **a** phylum, **b** family and **c** genus levels. **Figure S3.** Heatmap of relative abundance of SEED level 1 subsystems based on whole metagenome sequencing data. The e-value cutoff for metagenomics sequence matches to the SEED subsystem database was 1 × 10^− 5^ with a minimum alignment length of 15 amino acids. The two-way hierarchical cluster analysis was performed using unweighted pair group method with arithmetic mean (UPGMA) method. The side colors in the heatmap depicts the clustering of the subsystems based on the relative abundance. The yellow cluster indicate the SEED Subsystem with relative abundance above 7% while the green cluster represent the SEED Subsystem with relative abundance below 5%. **Figure S4.** Differences in the relative abundance of level 1SEED subsystems that were mapped to “Carbohydrates”, “Amino Acids and Derivatives”, “Stress Response” and “Virulence, Disease and Defense”. Corrected *P*-values are calculated using the Benjamini-Hochberg false discovery rate approach (*P* < 0.05). **Figure S5.** Comparison functional categories assigned to **a** “Virulence, Disease and Defense” SEED subsystem level 2 and **b** “Resistance to Antibiotics” SEED subsystem level 3 between nursing and weaned piglets based on whole metagenome shotgun sequences analyzed using MG-RAST. The error bars show the calculated standard deviation of four replicates, and the [*P* < 0.001], [*P* < 0.01] and [P < 0.05] were indicated as [***], [**] and [*], respectively. (PPTX 911 kb)

## Abbreviations

16S rRNA: 16S ribosomal ribonucleic acid
ANOSIM: Analysis of similarities
DNA: Deoxyribonucleic acid
dNTPs: Deoxynucleotide triphosphates
GIT: Gastrointestinal tract
HSP: Heat shock protein
MGRAST: Metagenomics rapid annotation using subsystem technology
NRC: National research council
OTU: Operational taxonomic unit
PCoA: Principal co-ordinates analysis
PCR: Polymerase chain reaction
PD: Phylogenetic diversity
QIIME: Quantitative insights into microbial ecology
ROS: Reactive oxygen species
STAMP: Statistical analysis of metagenomic profiles
