# Supplementary material for: The dynamics of the piglet gut microbiome during the weaning transition in association with health and nutrition
Source: J Anim Sci Biotechnol. 2018 Jul 30;9:54. doi: 10.1186/s40104-018-0269-6 (PMC6065057; doi:10.1186/s40104-018-0269-6)
Supplement: Supplementary file 1 — Table S1. Number of 16S rRNA gene sequence reads of nursing and weaned piglet fecal microbiota before and after quality control. Table S2. Summary of whole metagenome sequence data before and after quality control and annotation. (DOCX 18 kb) [file 40104_2018_269_MOESM1_ESM.docx]

| **Sample ID** | **Number of sequence reads** | | **QC-passed**  **Reads, %** |
| --- | --- | --- | --- |
|  | **Pre-QC** | **Post-QC** |  |
| N1 | 213,258 | 127,441 | 59.76 |
| N2 | 153,391 | 85,505 | 55.74 |
| N3 | 121,607 | 66,610 | 54.77 |
| N4 | 33,718 | 19,505 | 57.85 |
| N5 | 191,647 | 102,930 | 53.71 |
| N6 | 62,555 | 34,855 | 55.72 |
| N7 | 132,306 | 69,654 | 52.65 |
| N8 | 93,778 | 47,296 | 50.43 |
| N9 | 107,115 | 59,629 | 55.67 |
| N10 | 47,181 | 27,234 | 57.72 |
| W1 | 209,544 | 130,339 | 62.20 |
| W2 | 196,086 | 98,300 | 50.13 |
| W3 | 173,574 | 98,581 | 56.79 |
| W4 | 109,354 | 66,321 | 60.65 |
| W5 | 284,757 | 178,198 | 62.58 |
| W6 | 343,035 | 188,353 | 54.91 |
| W7 | 264,609 | 149,527 | 56.51 |
| W8 | 325,309 | 176,947 | 54.39 |
| W9 | 215,548 | 124,950 | 57.97 |
| W10 | 163,951 | 95,661 | 58.35 |

**Additional file 1: Table S1**. Number of 16S rRNA gene sequence reads of nursing and weaned piglet fecal microbiota before and after quality control.

^QC – quality control; N – nursing; W – weaned^

^The numbers followed by each letter N and W indicate the piglet number.^

**Additional file 1: Table S2** Summary of whole metagenome sequence data before and after quality control and annotation

| Sample ID | MG-RAST ID | No. of sequence reads | | Length of contigs, bp | | No. of contigs | | Alignment: Identified Protein Features | Annotation: Identified Functional Categories |
| --- | --- | --- | --- | --- | --- | --- | --- | --- | --- |
|  |  | Pre-QC | Post-QC | Pre-QC | Post-QC | Pre-QC | Post-QC |  |  |
| N1 | mgm4745743.3 | 4,531,548 | 4,530,646 | 74,800,360 | 52,416,370 | 81,447 | 79,410 | 55,257 | 31,527 |
| N2 | mgm4745747.3 | 6,870,302 | 6,868,798 | 130,241,602 | 103,118,418 | 164,528 | 160,633 | 103,541 | 61,547 |
| N3 | mgm4745746.3 | 4,288,774 | 4,287,375 | 69,391,125 | 53,568,785 | 81,677 | 79,941 | 62,874 | 36,796 |
| N4 | mgm4745740.3 | 4,654,022 | 4,653,262 | 82,589,526 | 66,491,857 | 108,853 | 106,095 | 71,088 | 40,136 |
| W1 | mgm4745745.3 | 9,447,406 | 9,445,251 | 151,116,638 | 113,319,181 | 179,364 | 165,774 | 98,920 | 44,941 |
| W2 | mgm4745744.3 | 7,113,170 | 7,111,597 | 151,002,242 | 112,874,763 | 161,617 | 155,573 | 105,330 | 55,088 |
| W3 | mgm4745741.3 | 6,320,228 | 6,318,750 | 144,162,529 | 106,003,305 | 165,591 | 160,988 | 104,264 | 56,570 |
| W4 | mgm4745742.3 | 7,215,282 | 7,213,515 | 173,768,565 | 131,398,512 | 224,894 | 212,007 | 132,418 | 67,400 |

^QC – quality control; N – nursing; W – weaned^

^The numbers followed by each letter N and W indicate the piglet number.^
