# Supplementary material for: The dynamics of the piglet gut microbiome during the weaning transition in association with health and nutrition
Source: J Anim Sci Biotechnol. 2018 Jul 30;9:54. doi: 10.1186/s40104-018-0269-6 (PMC6065057; doi:10.1186/s40104-018-0269-6)
Supplement: Supplementary file 2 — Figure S1. Extended error bar plot identifying the significantly different taxa between nursing and weaned piglets at the phylum (a), family (b) and genus (c) levels. Corrected P values are shown at right. The differences in the microbial community structure were measured using a two-sided Welch’s t-test, and P < 0.05 was considered significant. Figure S2. Comparison of the taxonomic profiles obtained using 16S rRNA gene and whole metagenome sequencing between nursing and weaned piglets. Stacked bar plots show the relative abundance of bacteria at the a phylum, b family and c genus levels. Figure S3. Heatmap of relative abundance of SEED level 1 subsystems based on whole metagenome sequencing data. The e-value cutoff for metagenomics sequence matches to the SEED subsystem database was 1 × 10− 5 with a minimum alignment length of 15 amino acids. The two-way hierarchical cluster analysis was performed using unweighted pair group method with arithmetic mean (UPGMA) method. The side colors in the heatmap depicts the clustering of the subsystems based on the relative abundance. The yellow cluster indicate the SEED Subsystem with relative abundance above 7% while the green cluster represent the SEED Subsystem with relative abundance below 5%. Figure S4. Differences in the relative abundance of level 1SEED subsystems that were mapped to “Carbohydrates”, “Amino Acids and Derivatives”, “Stress Response” and “Virulence, Disease and Defense”. Corrected P-values are calculated using the Benjamini-Hochberg false discovery rate approach (P < 0.05). Figure S5. Comparison functional categories assigned to a “Virulence, Disease and Defense” SEED subsystem level 2 and b “Resistance to Antibiotics” SEED subsystem level 3 between nursing and weaned piglets based on whole metagenome shotgun sequences analyzed using MG-RAST. The error bars show the calculated standard deviation of four replicates, and the [P < 0.001], [P < 0.01] and [P < 0.05] were indicated as [***], [**] and [*], respe [file 40104_2018_269_MOESM2_ESM.pptx]

## Slide 1
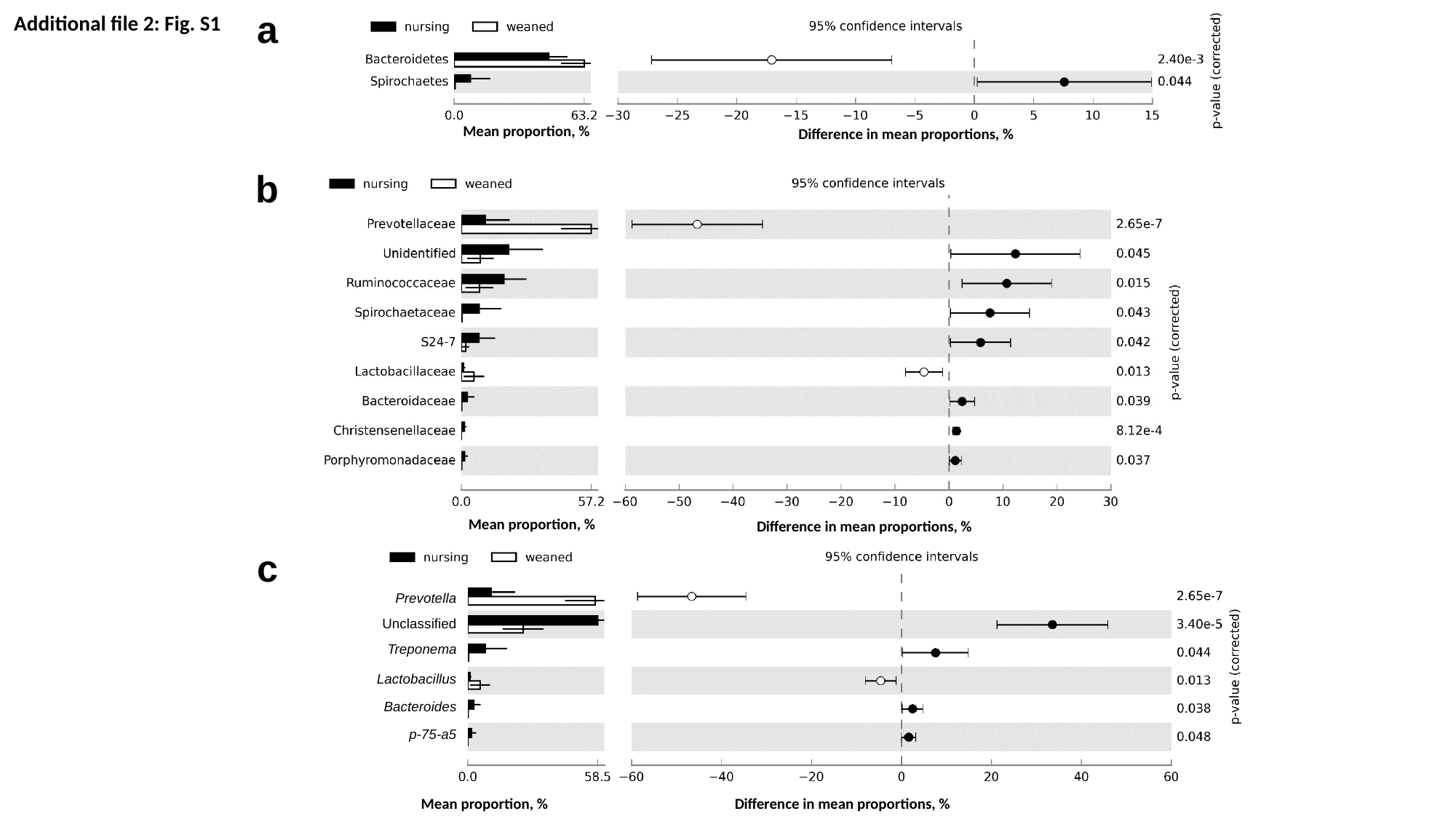

a
Additional file 2: Fig. S1
Mean proportion, %
Difference in mean proportions, %
b
Mean proportion, %
Difference in mean proportions, %
c
Prevotella
Unclassified
Treponema
Lactobacillus
Bacteroides
p-75-a5
Difference in mean proportions, %
Mean proportion, %

## Slide 2
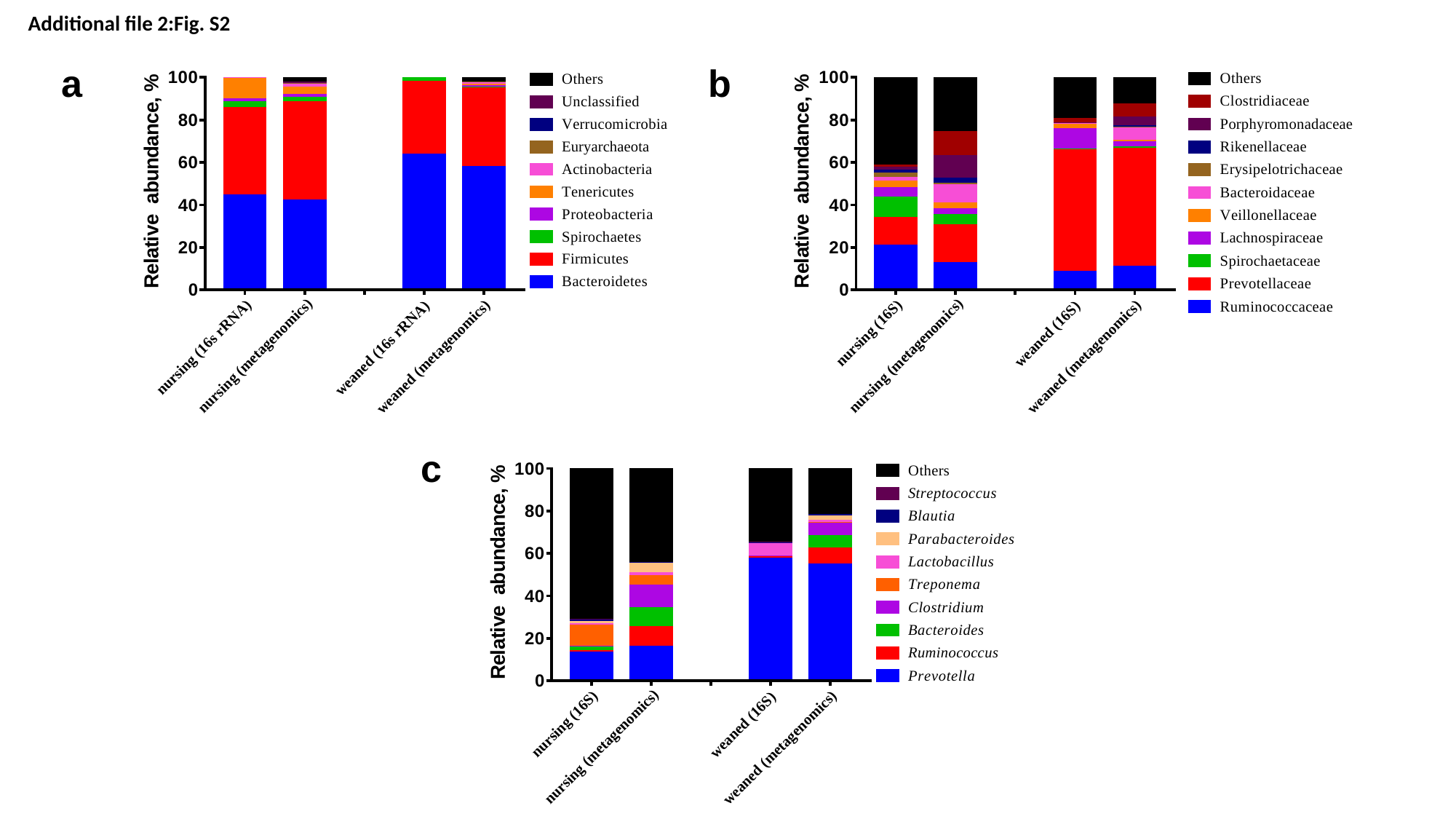

Additional file 2:Fig. S2
a
b
c

## Slide 3
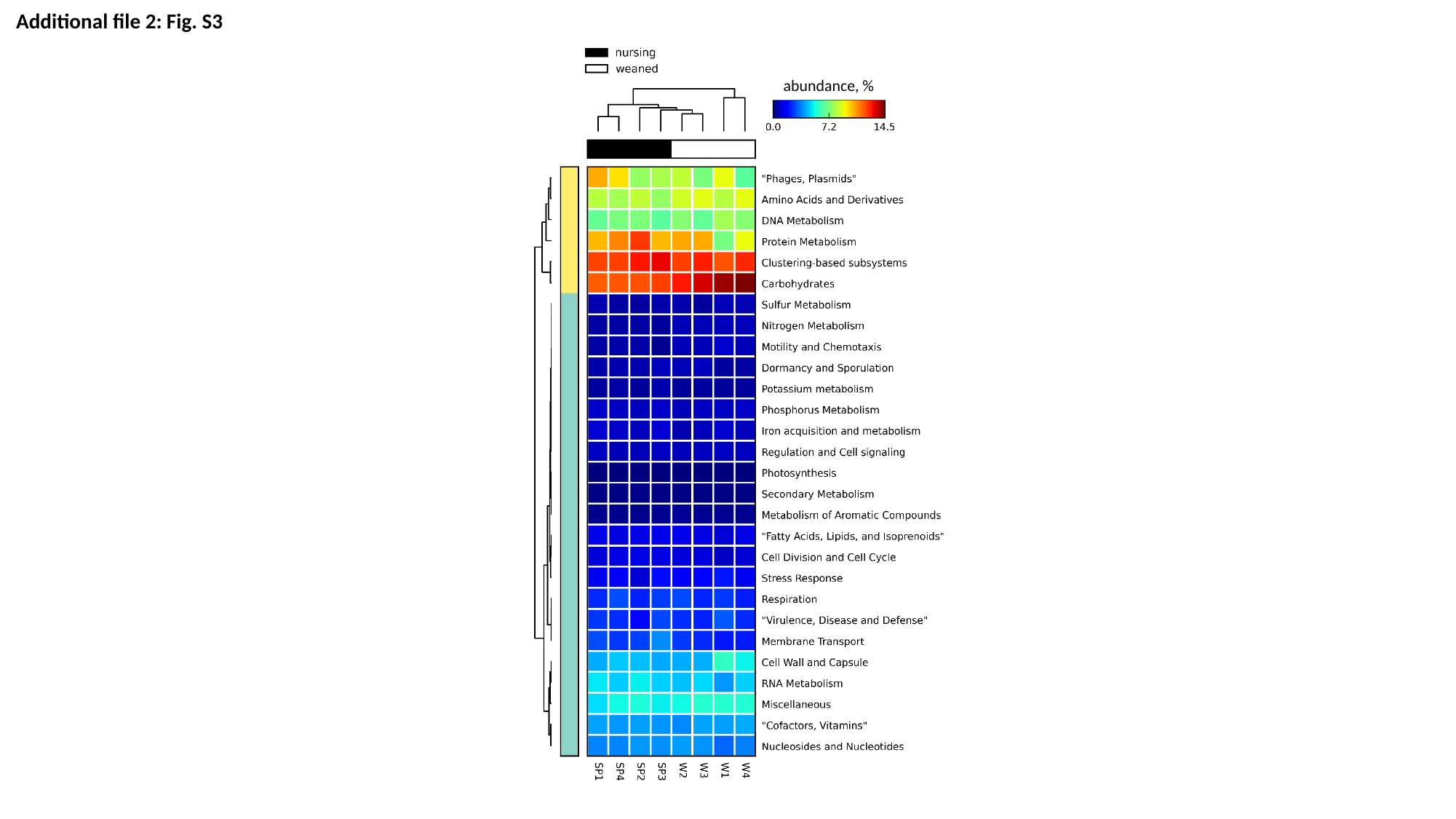

Additional file 2: Fig. S3
abundance, %

## Slide 4
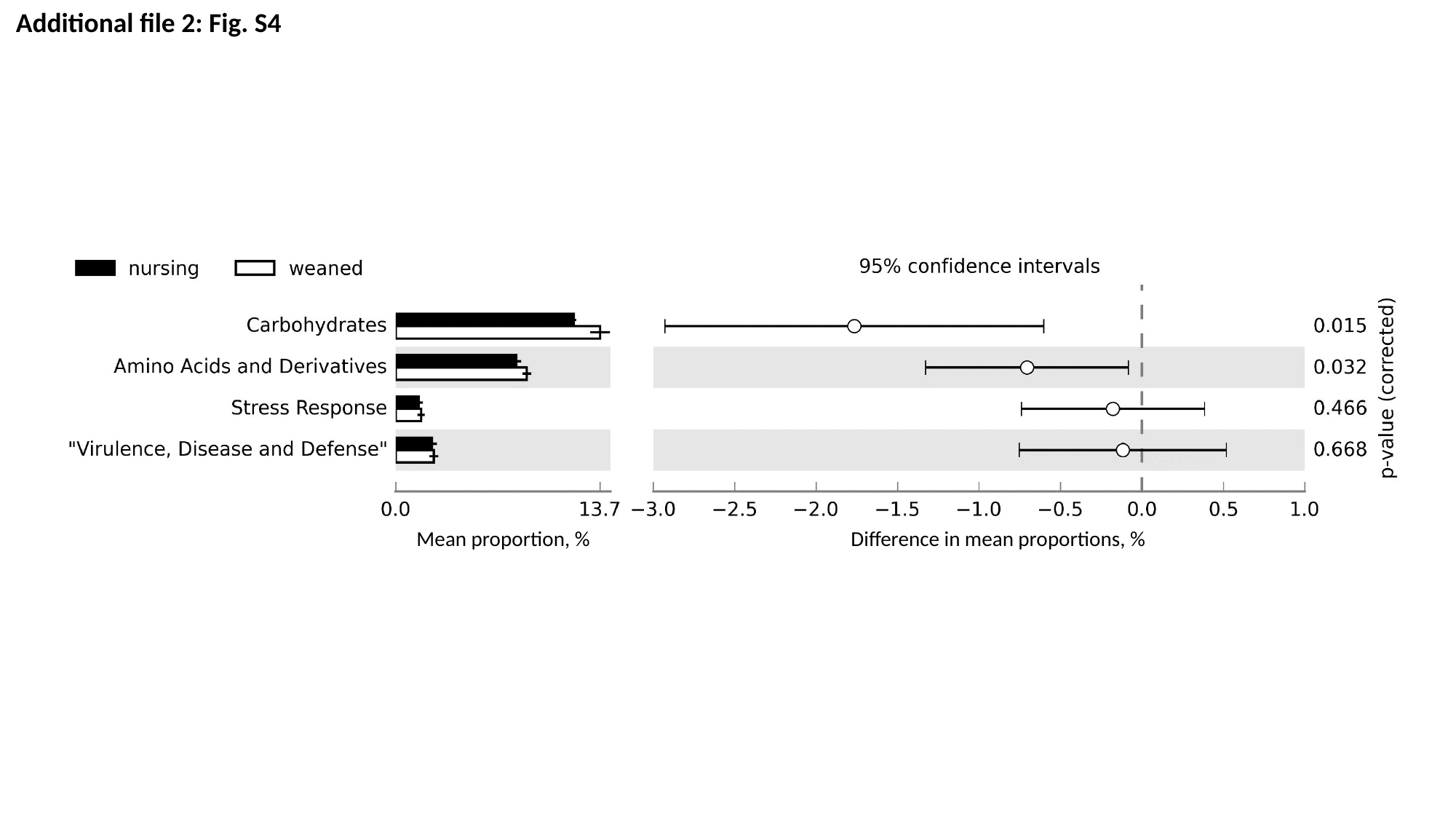

Additional file 2: Fig. S4
Difference in mean proportions, %
Mean proportion, %

## Slide 5
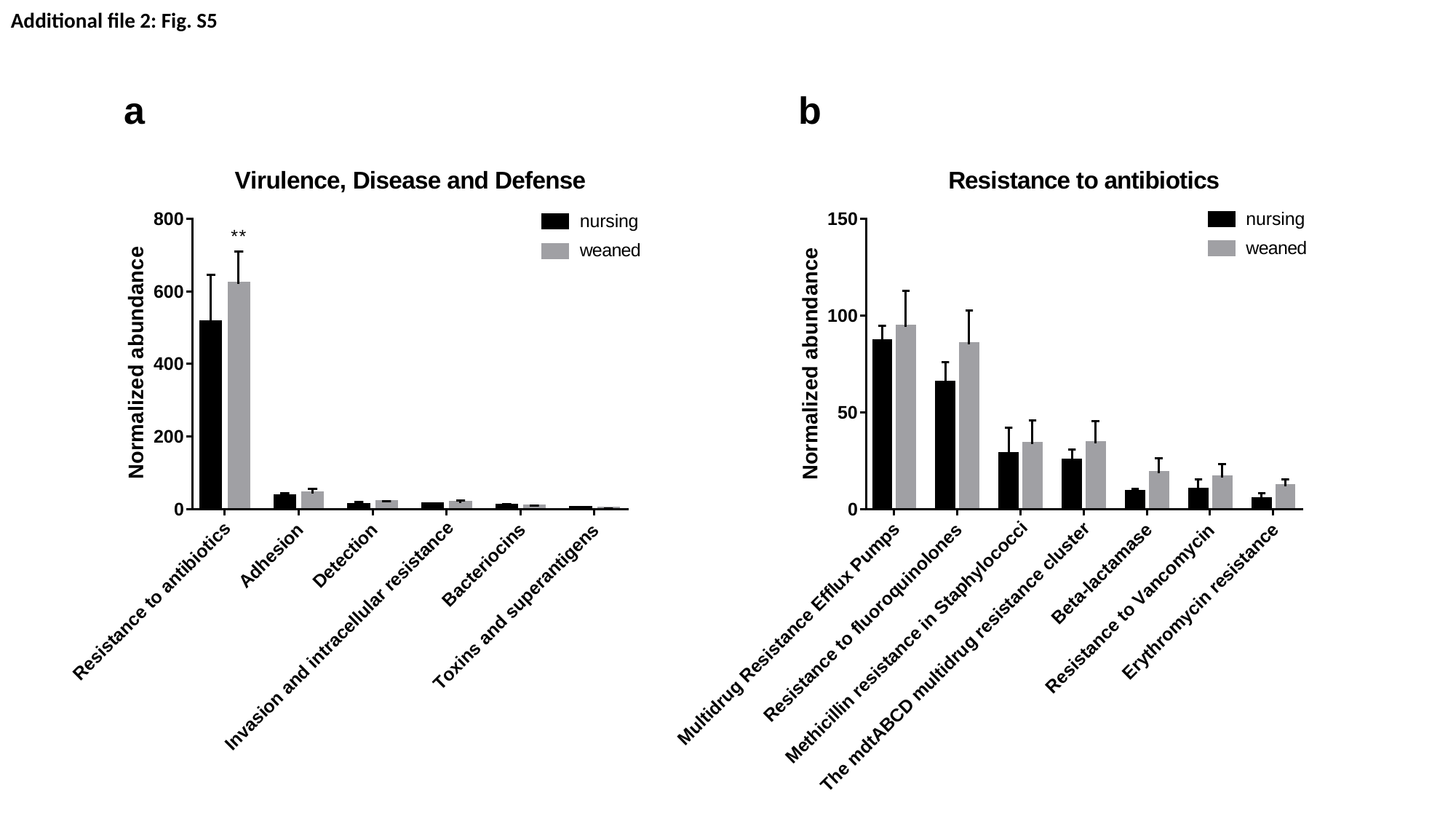

Additional file 2: Fig. S5
a
b
